# Optimizing the connection of CRRT and ECMO lines with additional pressure regulator on the therapeutic effect, filter life, and incidence of complications

**DOI:** 10.1097/MD.0000000000038580

**Published:** 2024-06-21

**Authors:** Xihua Huang, Yongxia Gao, Xufeng Chen, Yong Mei, Hui Zhang, Yali Tian, Juan Wu

**Affiliations:** a The First Affiliated Hospital of Nanjing Medical University (Jiangsu Province Hospital), EICU (Emergency Intensive Care Unit), Nanjing, Jiangsu, China.

**Keywords:** additional pressure regulator, CRRT, ECMO

## Abstract

**Background:**

Extracorporeal membrane oxygenation (ECMO) is used for severe cardiopulmonary failure, with veno-arterial ECMO for cardiogenic shock and veno-venous ECMO for acute respiratory failure. ECMO’s application has expanded to ICUs, emergency departments, and operating rooms. ECMO patients are at high risk for complications, including acute kidney injury (AKI), often requiring renal replacement therapy (RRT), posing significant management challenges.

**Methods:**

From August 2015 to June 2022, 120 patients were cured with veno-venous ECMO (n = 60) or veno-arterial ECMO (VA-ECMO, n = 60) combined with CRRT in our hospital. In the control group (n = 60), the input end (arterial end) of CRRT was connected to the ECMO oxygenator. The reinfusion end (venous end) of CRRT was connected to the oxygenator of ECMO for CRRT + ECMO treatment. In the experimental group (n = 60), the input end (arterial end) of CRRT was connected to the oxygenator of ECMO, and an additional pressure regulating device was installed on the connection of the 2 lines. The observation indexes including clinical therapeutic effect, clinical therapeutic effect, the incidence of complications, and the incidence of complications were compared.

**Results:**

There was a notable decrease in serum creatinine, and the differences in blood urea nitrogen, procalcitonin, and C-reactive protein after operation were statistically significant (*P* < .05). The filter use time in the study group was notably longer (*P* < .01). There exhibited no remarkable difference in the incidences of bleeding, thrombosis, numbness of hands and feet, metabolic alkalosis, disseminated intravascular coagulation, organ dysfunction syndrome, hyperbilirubinemia, and infection.

**Conclusion:**

This study demonstrates that additional pressure regulation devices are installed at the line connection between the CRRT input end and the CRRT return end to ensure that the flow rate of ECMO does not affect the CRRT treatment. ECMO and CRRT provide a safe pressure range so that the ECMO line can be safely connected to the CRRT machine at physiological pressure, reducing the occurrence of complications related to CRRT machine interruption and improving the efficiency of CRRT without affecting the efficiency of ECMO, ensuring patient safety.

## 1. Introduction

When a patient suffers from severe cardiopulmonary failure, extracorporeal membrane oxygenation (ECMO) is a recognized life-saving procedure.^[1,2]^ Refractory cardiogenic shock or other forms of severe shock or cardiopulmonary failure can be treated with veno-arterial ECMO. If hypoxemia or hyperthyroidism is present with a potentially reversible acute respiratory failure, veno-venous ECMO may be indicated.^[3]^ Due to the expanding range of indications for ECMO, its application has been enhanced in the intensive care unit as well as during emergency departments, operating rooms, cardiac catheterization laboratories, and interhospital transfers.^[4]^ The patients who use ECMO generally have inflammatory hemodynamic abnormalities, inflammation, and pathophysiological abnormalities of various organs, which puts them at high risk of other organ failure.^[4]^ The term “hyperthyroidism” is strange within the context of respiratory failure. The correct term is most likely “hypercapnia,” which clearly indicates increased amounts of carbon dioxide in the bloodstream and is directly related to respiratory failure. Due to the expanding range of ECMO indications, its application has increased the activity in intensive care units, emergency departments, operating rooms, cardiac catheterization laboratories, and inter-hospital transfers. Patients on ECMO support have inflammatory, hemodynamic, and pathophysiological abnormalities, which put them at risk for potential complications.^[4]^ According to current literature, 20% to 100% of ECMO acute renal injury (AKI) patients receive renal replacement therapy (RRT) before or during ECMO support. Approximately 85% of patients suffer from AKI. The reasons for AKI on ECMO are usually multifactorial.^[5]^ Causal factors include inflammation, hemodynamic instability, ischemia-reperfusion injury, abnormal platelet and coagulation function, and exposure to nephrotoxic substances. Also directly relevant to ECMO are factors such as red blood cell stress, hemolysis, and free iron release.^[6]^ECMO patients can develop AKI if they have excessive fluid infusions and fluid overload before and during the procedure. Twenty to one hundred percent of ECMOAKI patients receive RRT before or during their ECMO treatment, according to the current literature.^[7]^

Continuous RRT (CRRT) is the most commonly adopted method.^[8]^ Combining ECMO and CRRT remains a challenge.^[9]^ An integrated RRT system (ECMO system with RRT circuits integrated within it) or a parallel system (ECMO system with separate circuits) can be used during ECMO.^[10]^ These 2 modes provide all RRT modes, including continuous veno-venous hemofiltration, continuous veno-venous hemodialysis, continuous veno-venous hemodiafiltration, and slow continuous ultrafiltration.^[11]^ The CRRT can only be connected to the venous pressure from 0 to 20 mm Hg, while the pressure in the ECMO circuit is notably negative in front of the pump (range: −20 to −100 mm Hg). In addition, the pressure between the pump and the oxygenator is positive, which may exceed the normal physiological range of the CRRT device, resulting in CRRT interruption.^[12]^ In the meanwhile, the excessive negative pressure in front of the pump in the ECMO circulation pipe can lead to an air embolism in the ECMO circulation line caused by the entry of CRRT equipment into the air at the same time.^[13]^ In recent years, additional pressure devices have been introduced to optimize the connection between CRRT and ECMO lines in clinical practice whose application effect has been praised by medical staff and patients. However, there are only a few research reports that still lack solid scientific proof. Further research on this subject is awaited.

Stress management of CRRT lines is a globally recognized challenge. Pressure alarms can be suppressed or ECMO blood flow reduced to bypass high pressure, but the consequences are unpredictable. In recent years, additional pressure devices have been introduced to optimize the connection between CRRT and ECMO lines in clinics, whose application effect has been praised by many medical staff and patients. However, there are few such research reports, whose application effect still lacks scientific demonstration. It is very necessary to carry out related research under this background.

## 2. Patients and methods

### 2.1. General information

From August 2015 to June 2022, 120 patients were supported with veno-venous ECMO (n = 60) or veno-arterial ECMO (V-A ECMO, n = 60) combined with CRRT in our hospital. In the control group (n = 60), the input end (arterial end) of CRRT was connected to the ECMO oxygenator. The reinfusion end (venous end) of CRRT was connected to the oxygenator of ECMO for CRRT + ECMO treatment. In the experimental group (n = 60), the input end (arterial end) of CRRT was connected to the oxygenator of ECMO, and an additional pressure regulating device was installed on the connection of the 2 lines. There exhibited no notable difference in sex, age, and course of disease (*P* > .05), as indicated in Table 1.

**Table 1:** The general data in 2 groups.

Project	R group(n = 60)	C group (n = 60)	*χ*^2^/t	*P*
Age (y)	68.36 ± 4.22	69.42 ± 4.17	1.384	.169
Male， n (%)	33 (55.00)	30 (50.00)	0.301	.583
body mass index (BMI) (kg/m^2^)	24.42 ± 3.40	24.16 ± 3.35	0.422	.674
Basal body temperature (°C)	37.70 ± 0.99	37.58 ± 0.11	0.933	.353
Number of y of education (y)	8.43 ± 1.22	8.50 ± 1.24	0.312	.756
APACHE Ⅱ scoring (points, x ± s)	23.23 ± 4.12	24.14 ± 3.31	1.334	.185
ECMO running time (h,x ± s)	118.34 ± 12.54	114.78 ± 11.87	1.597	.113
CRRT Running time (h,x ± s)	99.26 ± 8.36	97.36 ± 7.79	1.288	.200
Primary disease			0.294	.592
Fulminant myocarditis (%)	5 (8.3)	4 (6.6)		
Severe acute respiratory distress syndrome (%)	15 (25)	14 (23.3)		
Multiple organ failure (%)	7 (11.6)	8 (13.3)		
Intractable heart failure (%)	13 (21.6)	14 (0.23)		
Hemorrhagic fever with renal syndrome (%)	4 (6.6)	3 (5)		
Acute renal failure (%)	16 (26.6)	17 (28.3)		

APACHEII = acute physiology and chronic health score Ⅱ; ECMO = extracorporeal membrane oxygenation; CRRT = continuous renal replacement therapy.

Inclusion criteria:

1. The age of the patient was ≥ 18 years old;
2. According to Berlin definition, it was diagnosed as severe acute respiratory distress syndrome ^[14]^; complicated with sepsis^[15]^ and multiple organ failure,^[16]^ ECMO combined with CRRT was needed.
3. The patient has the applicable conditions of ECMO combined with CRRT treatment indication and additional pressure-regulating device technology. ECMO combined with CRRT treatment indication was as follows. The patient had no respiratory system and other viral diseases in the past, and the heart function of the patient was damaged after onset, so circulatory and respiratory support was needed. The applicable conditions of the additional pressure regulating device technology showed there was no clear restriction on the use of this technology. All subjects were included to match the time of observation.
4. The scores of acute physiology and chronic health status were ≥ 15.
5. Vein-vein ECMO or veno-arterial ECMO treatment mode was used.
6. No heart, liver, kidney, or other medical complications.
7. Conscious patients, voluntarily participated in this study and were able to complete the follow-up successfully.

Exclusion criteria:

1. The treatment time of EMCO combined with CRRT was <72 hours.
2. The age of the patient was <18 years old.
3. The patients with chronic renal failure needed long-term dialysis treatment.
4. A person who was in a permanent coma after cardiac arrest.
5. Severe blood clotting abnormalities.
6. The patient was suffering from notable impairment of consciousness, and mental illness, and was unable to communicate normally.
7. The family gave up treatment halfway.
8. There were serious irreversible diseases, such as those who died within 24 hours after ECMO was on the machine.
9. Insufficient or missing data related to ECMO or CRRT.

### 2.2. Treatment methods

#### 2.2.1. Technical route

**Figure 1.**
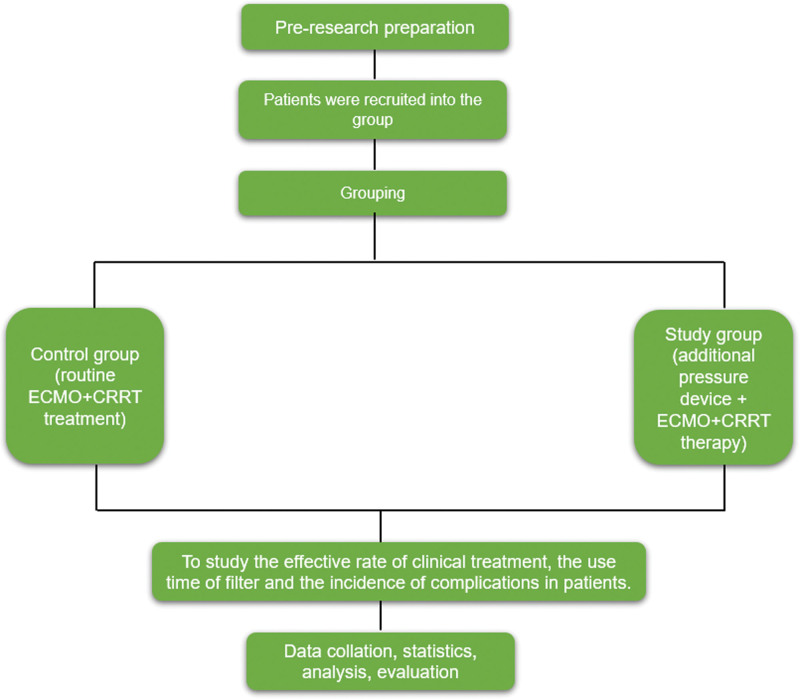
Technology roadmap.

The technical route is shown in Figure 1.

#### 2.2.2. Intervention scheme

In the experimental group (n = 60), the input end (arterial end) of CRRT was connected to the oxygenator of ECMO, and an additional pressure regulating device was installed on the connection of the 2 lines. In the control group, the input end (arterial end) of CRRT was connected to ECMO oxygenator, and the reinfusion end (venous end) of CRRT was connected to the oxygenator of ECMO for CRRT + ECMO treatment.

**Figure 2.**
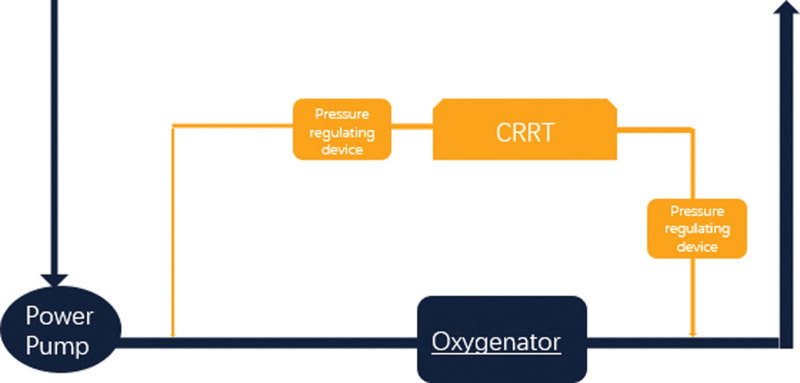
Schematic diagram of additional pressure regulating device.

In the experimental group, the input end of CRRT (arterial end) was connected to the oxygenator of ECMO. An additional pressure-regulating device was installed on the connection of the 2 lines. The return end (intravenous end) of the CRRT was linked to the ECMO oxygenator and an additional pressure regulator was installed at the connection of the 2 lines. As indicated in Figure 2.

### 2.3. Observation index

1. Clinical therapeutic effect: clinical indices of serum creatinine, blood urea nitrogen, calcitonin, and C-reactive protein before and 72 hours after treatment in both groups.
2. Filter usage time. Filter usage time refers to the time difference between the end of filter use and the start time.
3. The incidence of complications: the number and rate of complications such as bleeding, venous thrombosis, hand and foot numbness, metabolic alkalosis, and so on were counted during and within 2 weeks after treatment.
4. The pressure of CRRT blood return end: the pressure value of the CRRT blood return end was monitored and collected in real-time through the pressure cap of the CRRT pipeline and the pressure detector of the equipment.

### 2.4. Statistical analysis

The data were analyzed using SPSS24.0 software. Mean ± standard deviation (x ± s) was used for measurements with a normal distribution and uniform variance, intergroup comparisons were performed using independent samples t-tests, and intragroup comparisons were conducted using paired samples t-tests. The counting data were expressed by frequency and percentage (%), and the comparison between groups was performed by *χ*^2^ test. *P* < .05 was regarded as statistically notable.

### 2.5. Ethical approval

The Ethical Committee of the hospital approved the study method and the consent from each patient was duly obtained.

## 3. Results

### 3.1. General data comparison

There was no significant difference in sex, age, and course of disease (*P* > .05) as shown in Table 1. Table 1 presents a comprehensive examination of the demographic and clinical attributes of 2 groups, Project R (n = 60) and Project C (n = 60). The age distributions of the groups are comparable, with mean ages of 68.36 ± 4.22 and 69.42 ± 4.17 years, respectively, indicating no statistically significant difference (t = 1.384, *P* = .169). In Project R, the male percentage is 55.00%, whereas in Project C it is 50.00%. There is no significant difference between the 2 proportions, as indicated by a *χ*^2^ value of 0.301 and a *P* value of .583. No statistically significant differences were found between the 2 groups in terms of other characteristics such as body mass index, basal body temperature, years of education, APACHE II scoring, ECMO running time, and CRRT running time. The distribution of primary diseases is shown, and although the percentages of particular disease categories differ, the overall distribution indicates that there is no significant difference between Project R and Project C (*χ*^2^ = 0.294, *P* = .592). Based on the above data, it can be concluded that the 2 groups are similar regarding several demographic and clinical factors.

### 3.2. The therapeutic effects between the 2 groups

There was no significant difference in serum creatinine, blood urea nitrogen, procalcitonin, and C-reactive protein before operation (*P* > .05). The levels of serum creatinine, blood urea nitrogen, procalcitonin, and C-reactive protein decreased notably after operation in the control and research groups (*P* < .05, Table 2).

**Table 2:** The alterations of various indexes pre- and post-treatment.

Project	R group (n = 60)				C group (n = 60)			
	Before treatment	After treatment	t	*P*	Before treatment	After treatment	t	*P*
Serum creatinine (mmol/L)	39.90 ± 2.32	15.93 ± 2.10	59.333	<.001	38.01 ± 1.50*	15.80 ± 1.41**	83.568	<.001
Urea nitrogen (μmol/L)	273.41 ± 15.32	112.24 ± 4.72	77.877	<.001	281.16 ± 12.30*	109.61 ± 4.16**	102.339	<.001
Procalcitonin (μg/L)	4.54 ± 0.14	2.31 ± 0.42	39.017	<.001	4.71 ± 0.23*	2.21 ± 0.13**	73.297	<.001
C Reactive protein (mg/L)	181.17 ± 8.20	54.32 ± 2.12	116.012	<.001	178.71 ± 6.50*	54.46 ± 2.42**	138.874	<.001

* *P* > .05 indicated compared with the study group before treatment.

** *P* > .05 indicated compared with the study group after treatment.

### 3.3. Blood pressure of CRRT

When the ECMO blood flow rate was 3 L/min, the CRRT circuit pressure of the experimental group was 45.15 ± 1.74 mm Hg, and that of the control group was −69.00 ± 2.12 mm Hg. The difference had the significance of systematical calculation between the 2 groups (t = −262.889, *P* < .001). Table 3 shows the blood pressure recordings of the 2 groups.

**Table 3:** The blood pressure of CRRT.

	R group (n = 60)	C group (n = 60)	t	*P*
CRRT loop pressure (mm Hg)	45.15 ± 1.74	−69.00 ± 2.12	−262.889	＜.001

CRRT = continuous renal replacement therapy.

### 3.4. Comparison of usage time of filters

In terms of the filter use time, the filter use time in the study group was notably longer than that of the control group (*P* < .01, Table 4).

**Table 4:** The usage time of 2 sets of filters.

	R group (n = 60)	C group (n = 60)	t	*P*
Use time (h)	38.24 ± 4.12	27.12 ± 5.13	29.091	＜.01

### 3.5. Comparison of complications

There were no notable differences in the incidences of bleeding, thrombosis, numbness of hands and feet, metabolic alkalosis, disseminated intravascular coagulation, organ dysfunction syndrome, hyperbilirubinemia, and infection between the 2 groups (*P* > .05, Table 5).

**Table 5:** The incidence of complications.

Project	R group (n = 60)	C group (n = 60)	*χ* ^2^	*P*
Hemorrhage	6(10.00)	12(20.00)	2.353	.125
Thrombosis	2(3.33)	4(6.67)	0.702	.402
Numbness of hands and feet	19(31.67)	18(30.00)	0.039	.843
Metabolic alkalosis	1(1.67)	2(3.33)	0.342	.559
Disseminated intravascular coagulation	1(1.67)	2(3.33)	0.342	.559
Organ dysfunction syndrome	4(6.67)	2(3.33)	0.702	.402
Hyperbilirubinemia	6(10.00)	4(6.67)	0.436	.509
Infected	13(21.67)	15(25.00)	0.186	.666

## 4. Discussion

ECMO is a life-support machine for patients suffering from serious life-threatening illnesses, infections that cause severe lung damage, or heart attacks that result in shock.^[17]^ Plastic lines are inserted into large veins and arteries of the legs, neck, or chest to connect ECMO to the patient. Blood can be pumped from the patient body into the oxygenator (artificial lung). This will help remove carbon dioxide from the body of the patient, as well as add oxygen to it. As a result, this device^[18]^ has replaced one lungs. By replacing the heart with an ECMO machine, the blood is then pumped back into the patient body with the same force as the heart. Overloading patients with fluids is not well tolerated by those with severe cardiovascular disease or respiratory failure.^[19]^ CRRT replaces the kidney purification function through a slow and steady extracorporeal blood purification device. The aim has been to mildly correct fluid overload in patients and remove excessive uremic toxins.^[20]^ Patients with severe AKI who are critically ill are at high risk for death if left untreated. Many observational studies have suggested that CRRT is the main form of RRT in critically ill patients with AKI and/or multiple organ failure (usually caused by septic shock) in intensive care units, Acute brain injury or elevated intracranial pressure and systemic brain edema may also cause these conditions.^[21]^ In both adult and pediatric patients, CRRT is effective because it controls volume precisely, corrects acid-base balance, and maintains hemodynamic stability.^[22]^

During ECMO, ECMO is required in up to 50% of patients who need RRT.^[23]^ Most patients use RRT in combination to remove fluid and correct metabolic disorders.^[24]^ Hemodynamically unstable or acutely ill patients cannot tolerate rapid fluctuations in their fluid balance and metabolic rate.^[25]^ The advantage of CRRT is that it provides more consistent liquid removal over a longer period, less fluctuation, and possibly shorter ECMO duration. In addition, Intermittent hemodialysis often causes ECMO flow to be difficult to maintain, so patients and ECMO circuits are generally better able to tolerate slow fluid clearance of CRRT.^[26]^ The introduction of RRT can save lives; it also means increased complexity of care, increased risk of potential side effects, and increased medical costs. A meta-analysis included 43 observational studies, of which 21,624 patients who received ECMO showed higher mortality among ECMO patients who needed <30% of RRT. On the other hand, the same systematic review conducted in very heterogeneous studies showed that patients with combined ECMO and RRT had higher mortality rates than ECMO alone. The direct role of RRT in reducing the risk of death is uncertain. Most likely, the higher mortality rates observed are due to the effects of severe AKI.^[27]^ Although ECMO patients with AKI and severe volume overload, uremia, acid-base, and electrolyte imbalance should receive RRT, it is not clear whether ECMO + RRT can lead to better results.^[28]^

CRRT is routinely connected to the central venous hemodialysis catheter, so the operating pressure is usually 0 to 20 mm Hg. There are 3 pressure zones in the ECMO circulation line, including pre-centrifugal pump, pre-centrifugal pump-membrane lung, and post-membrane lung.^[29]^ When the positive or negative pressure produced by the blood flow in the ECMO pipeline exceeds the CRRT pressure alarm threshold, CRRT treatment may be interrupted. At present, there are 3 common connection methods for ECMO combined with CRRT. Firstly, the CRRT is connected to the dialysis catheter, the 2 sets of cardiopulmonary bypass systems operate independently, and the hemodynamics do not interfere with each other. However, the placement of CRRT catheters during systemic heparinization of ECMO may increase the incidence of bleeding and infection.^[30]^ In addition, the CRRT is connected to the ECMO pipe through the hemofilter. The draining end of the hemofilter is connected to the back of the ECMO centrifugal pump or the membrane lung, the blood return end of the hemofilter is connected in front of the ECMO centrifugal pump, and the infusion pump controls the flow rate of the ultrafiltrate.^[31]^ However, some studies have pointed out that there are errors in controlling the flow rate of ultrafiltrate with an infusion pump, and the pipeline pressure of the hemofilter cannot be monitored, which is prone to complications such as membrane rupture. Finally, the CRRT parallel ECMO pipeline does not need additional dialysis catheters, which can accurately control the treatment parameters and monitor the pressure values, but there is a problem of hemodynamic interference between ECMO and CRRT.^[32]^ The pressure levels in different sections of the ECMO circuit may not be compatible with the CRRT pressure threshold. Changing the ECMO blood flow will cause these pressure changes, thus changing the pressure in the CRRT line.^[33]^ Detection of pressure exceeding the alarm range will cause the CRRT device to stop. If the stress is not managed properly, the cessation of CRRT may reduce the useful life of CRRT. High pressures can be avoided by suppressing pressure alarms or reducing ECMO blood flow but may have unpredictable consequences.^[34]^

The results showed that the therapeutic effect of optimizing the CRRT and ECMO pipeline connections with the additional pressure-regulating device had no notable difference compared with the control group, which indicated that the application of this device would not affect the therapeutic effect of the patients. This is probably because the purpose of installing the additional pressure device is to regulate the pressure in the circuit, prevent the interruption of treatment, and prolong the life of the CRRT. After using the additional pressure-regulating device to optimize the CRRT and ECMO pipeline connections, the filter usage time was notably longer than that of the control group without the additional pressure-regulating device. After the application of the additional pressure-regulating device, the pressure in the pipeline can be well controlled. The continuity of treatment can be maintained, and the CRRT equipment can be prevented from being stopped due to excessive pressure. In the process of application, the CRRT machine did not report the alarm of excessive pressure. Because the pre-pump pressure is positive and the post-pump and pre-membrane pressures are negative, this is more in line with the pressure threshold during CRRT treatment. In this study, additional pressure regulators did not increase serious complications such as bleeding, thrombosis, and disseminated intravascular coagulation. It can be seen that the use of an additional pressure regulator to optimize the integrated support of ECMO combined with CRRT treatment will not increase the damage caused by complications, maximize the effectiveness of clinical treatment, and protect the life safety of patients. Compared with conventional nursing, nursing using additional pressure regulating devices to optimize the integrated support of ECMO combined with CRRT therapy has more complex operations and more monitoring indicators, and the professional quality of nursing staff is relatively higher. It can quickly evaluate the clinical indicators of patients, correctly deal with equipment warning problems, and provide a strong guarantee for the life and health of patients. However, there are limitations to the study. ECMO combined with CRRT is difficult as a novel treatment, with high costs, and there is a lack of uniform application and standards. The lack of large sample data support for clinical research is an urgent problem to be solved in the future.

In summary, this study compared the clinical therapeutic effect, clinical therapeutic effect, the incidence of complications, and the incidence of complications in different groups. As a result, the addition of a pressure-regulating device to the tubing connection at the CRRT input and CRRT return end can control the flow rate and pressure to ensure that the flow rate of ECMO does not interfere with the CRRT treatment. It provides a safe pressure range for ECMO and CRRT so that the ECMO tubing is safely connected to the CRRT machine at physiological pressure and reduces the occurrence of complications related to CRRT machine interruptions. It can also improve the efficiency of CRRT without affecting the efficiency of ECMO, prolonging the life of the filter and ensuring patient safety.

## Acknowledgments

We would like to extend our gratitude to all those who contributed to the successful completion of this research. Special thanks go to the staff of First Affiliated Hospital of Nanjing Medical University (Jiangsu Provincial People Hospital), for their invaluable assistance and support. We also appreciate the cooperation of the patients and their families who participated in this study. Furthermore, we acknowledge the contributions of our colleagues and institutions that provided critical feedback and resources.

## Author contributions

**Conceptualization:** Xihua Huang, Yongxia Gao, Juan Wu, Yong Mei, Yali Tian.

**Data curation:** Xihua Huang, Yongxia Gao, Xufeng Chen, Yong Mei, Hui Zhang, Yali Tian.

**Formal analysis:** Xihua Huang, Yongxia Gao, Yong Mei, Hui Zhang.

**Funding acquisition:** Xihua Huang, Juan Wu, Yongxia Gao.

**Investigation:** Xihua Huang, Xufeng Chen, Hui Zhang.

**Methodology:** Xihua Huang, Yongxia Gao, Yong Mei.

**Resources:** Xihua Huang, Xufeng Chen, Yali Tian.

**Software:** Xihua Huang, Xufeng Chen, Yongxia Gao.

**Supervision:** Xihua Huang, Juan Wu, Yong Mei.

**Validation:** Xihua Huang, Juan Wu, Xufeng Chen.

**Visualization:** Xihua Huang, Yongxia Gao, Hui Zhang.

**Writing – original draft:** Xihua Huang, Yongxia Gao, Xufeng Chen, Yali Tian.

**Writing – review & editing:** Xihua Huang, Juan Wu.
